# Phloem Sap Composition: What Have We Learnt from Metabolomics?

**DOI:** 10.3390/ijms24086917

**Published:** 2023-04-07

**Authors:** Louis Broussard, Cyril Abadie, Julie Lalande, Anis M. Limami, Jérémy Lothier, Guillaume Tcherkez

**Affiliations:** 1 Institut de Recherche en Horticulture et Semences, Université d’Angers, INRAe, 42 rue Georges Morel, 49070 Beaucouzé, France; 2 Research School of Biology, Australian National University, Canberra, ACT 2601, Australia

**Keywords:** phloem, sap, metabolome, metabolic cycle, sampling

## Abstract

Phloem sap transport is essential for plant nutrition and development since it mediates redistribution of nutrients, metabolites and signaling molecules. However, its biochemical composition is not so well-known because phloem sap sampling is difficult and does not always allow extensive chemical analysis. In the past years, efforts have been devoted to metabolomics analyses of phloem sap using either liquid chromatography or gas chromatography coupled with mass spectrometry. Phloem sap metabolomics is of importance to understand how metabolites can be exchanged between plant organs and how metabolite allocation may impact plant growth and development. Here, we provide an overview of our current knowledge of phloem sap metabolome and physiological information obtained therefrom. Although metabolomics analyses of phloem sap are still not numerous, they show that metabolites present in sap are not just sugars and amino acids but that many more metabolic pathways are represented. They further suggest that metabolite exchange between source and sink organs is a general phenomenon, offering opportunities for metabolic cycles at the whole-plant scale. Such cycles reflect metabolic interdependence of plant organs and shoot–root coordination of plant growth and development.

## 1. Introduction

Plant organs do not grow and operate separately since they are connected via long-distance transport systems (xylem and phloem). While xylem mostly conducts water and nutrients (along with organic molecules at low concentration, such as amino acids, iron-chelates or hormones) from roots to aboveground organs, phloem carries inorganic ions and many organic molecules coming from mature leaves, sustaining growth and development of heterotrophic organs, and from remobilized material, especially in perennials. In effect, in addition to playing a crucial role in allocation of photosynthates, phloem sap transport is crucial to communicate the nutritional status (shoot to root signaling), trigger defense responses or regulate developmental processes [1]. Specialized cells enable the phloem tissue to achieve this: sieve-tube elements (SE; non-lignified cellulosic transport tubes without nucleus, vacuoles, and ribosomes), companion cells (CC; supporting cells capable of gene expression and translation), and phloem parenchyma cells. CC and parenchyma cells are also crucial for (un)loading of transported molecules via plasmodesmata and specialization into transfer cells, respectively. Importantly, SE are connected via sieve plates (SP) coming from the selective hydrolysis of cell walls [2,3]. Phloem sap contains not only metabolites but also proteins (mostly P-proteins) and nucleic acids. P-Proteins may have several morphological forms (granular, filamentous or fibrillar, tubular, and crystalline) depending on developmental stage, environmental conditions and species. Filaments formed by P-proteins play several roles during cell specialization, and sap transport and P-protein aggregation is involved in the rapid sealing of SP pores [4,5].

This overview of phloem structure and function highlights key metabolic features of phloem sap transport (here, the term “phloem sap” refers to the circulating fluid in SE, and does not refer to total phloem fluid, i.e., contained by all cell types: CC, phloem parenchyma cells and SE; see also definitions in Supplementary Table S1). First, it redistributes sugars synthesized during photosynthesis from source organs (mature leaves) to sink tissues such as roots, flowers, fruits and seeds [6,7]. In general, sugars are at a very high concentration (hundreds of millimolar) and, combined with P-proteins, impacts sap viscosity. Second, signalling mediated by phloem sap may involve metabolites that are known to act as regulators or allosteric effectors, such as sucrose or hormones [1,7,8] (e.g., abscisic acid or cytokinins). Third, N- and S-containing molecules (amino acids and others) are also amongst phloem metabolites resulting from nitrate and sulphate assimilation in leaves [9,10,11,12,13]. For these reasons, metabolite transport via phloem sap is essential for plant growth and development, since it redistributes carbon, nitrogen and sulfur, and is involved in long-distance signalling.

Despite this importance, relatively little is known in terms of detailed and comprehensive metabolic composition of sap (i.e., sap metabolome). The major difficulty is our ability to collect phloem sap [14,15], not only to obtain a representative, unadulterated sample, but also to have sufficient material to carry out extensive analyses via nuclear magnetic resonance (NMR), gas chromatography/mass spectrometry (GC-MS) or liquid chromatography/mass spectrometry (LC-MS). Therefore, despite the considerable number of papers where phloem sap was analysed, referred to or reported (presently, about 25,000 articles can be retrieved on Google Scholar with “phloem sap” + “plant”, for example), our understanding of phloem sap metabolome is very limited. This represents a conundrum for plant physiology not only in terms of carbon, nitrogen and sulfur allocation within the plant, but also to understand the role of metabolites in plant signalling. In addition, metabolic composition is crucial to better understand how phloem sap circulates since metabolic composition impacts osmotic pressure, viscosity, ion coordination and P-protein aggregation and therefore sap velocity.

In this mini-review we shall focus on advances in knowledge of phloem sap metabolic composition via metabolomics. We will briefly address the following questions: (i) What methods can be used to collect sap samples for metabolomics? (ii) What are the key components of phloem sap metabolome? (iii) Does phloem sap metabolome relate to whole plant physiology, and does it respond to environmental cues? And (iv) could phloem sap transport participate in whole-plant metabolic exchanges? Information about transporters involved in loading metabolites into the phloem has been provided previously and will not be discussed here [16,17].

## 2. Phloem Sap Sampling Methods for Metabolomics: Overview

**Figure 1:**
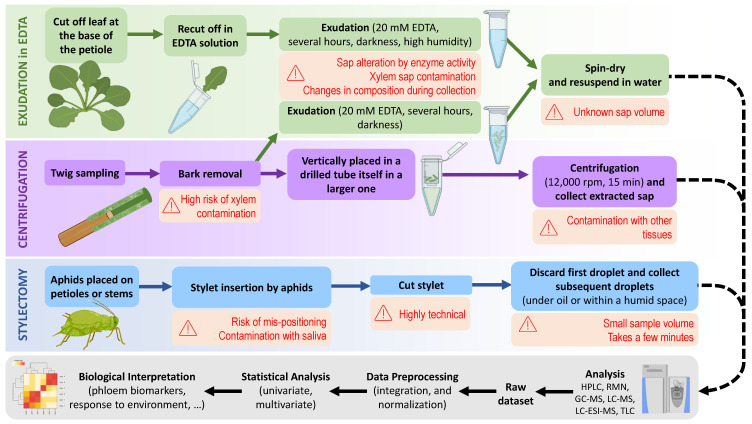
Summary chart of mainstream methods to sample and analyze phloem sap and potential biases associated with them (in red). See main text for further details.

The difficulty of accessing SE directly is one of the major barriers to analysing phloem sap. In fact, SE are extremely sensitive to physical injuries and wounding and are capable of effective sealing mechanisms, involving P-protein aggregation and/or callose deposition. To date, two mainstream methods (summarized in Figure 1) have been used to collect phloem sap: the aphid stylet method (stylectomy), and exudation in ethylenediaminetetraacetic acid (EDTA) solution (EDTA method reviewed in [18]). Direct phloem sap bleeding after incision can also be used in specific species, such as castor beans (*Ricinus communis*) [19], unlike the vast majority of other plant species [20]. As a matter of fact, many data on phloem sap composition have been obtained in this species (reviewed in [21]). Cucurbits (*Cucurbita* sp.) have also been used for direct exudation after incision, but the exudated fluid comes from external phloem which is not strictly representative of conducting phloem from the stele, including for the metabolome [22]. Sampling sap under the microscope with microcapillaries has also been proposed, combined with SE localization using phloem-mobile fluorochromes such as carboxy-fluorescein diacetate (see, e.g., [23,24]). Phloem cells can also be isolated using laser microdissection [25,26,27] or can be fluorescence-tagged and cell-sorted [28,29]. Interestingly, SE can also be followed using isotopic labelling in metabolites (^11^C or ^14^C photosynthates) [30,31,32]. However, microscopic and histological methods are implementable at low through-put, are limited to specific (sub)cellular studies and are not amenable at a broader scale to metabolic analysis, so they will not be discussed further.

The aphid stylet method has been set up a long time ago [33]. It consists of cutting the stylet of a sap-feeding insect (aphid species) during feeding and collecting the exudate with a microcapillary [34]. Despite its frequent use on grass species and cereals, and occasional utilization in legumes, this approach is generally hard to use on dicotyledonous plants [35]. It is also highly intricate and usually allows collection of small sap volumes. It was believed that phloem sap collected via the aphid-stylet method was pure. However, there can be some mixing with proteins and other components coming from insect saliva. Additionally, many phloem sap-feeders occasionally consume xylem sap, and thus the aphid stylet method can lead to a mixture of xylem and phloem saps [36]. However, it is possible to determine tissues effectively punctured by aphids using electrical penetration graph (EPG), which allows one to monitor the different steps of aphid behavior (penetration, salivation, puncture and ingestion) [37,38,39]. Specific electrical signals in EPG reflect feeding activity of the aphid [40,41] including xylem puncture [42], and thus can be used to determine the feeding status at the time of stylectomy [43].

EDTA (usually in the K^+^ form, K_2_-EDTA) is used for chelator-facilitated exudation. Exudation in the EDTA solution is adapted to cut petiole tips or pieces of bark and avoids callose and P-protein aggregation. This technique enables easy phloem sap sampling from most plant species and in large amounts [44,45]. However, the EDTA method is problematic for several reasons: (i) there could be contaminations with xylem sap and fluids from other tissues favoured by cell wall loosening via the chelating action of EDTA [46]; (ii) enzymatic activities can change sap composition considerably in particular due to enzymes hardly affected by EDTA such as invertases; (iii) there can be some reabsorption of exuded compounds when transpiration is not totally suppressed by saturating humidity; (iv) exudation requires a long time (about 8 h in standard protocols for *Arabidopsis thaliana*; hereafter abbreviated *Arabidopsis*), and thus there can be considerable changes in composition with time; and (v) exudated sap volume is generally unknown since it is very small compared to that of the EDTA solution, and therefore the actual metabolite concentration in the sap is unknown. An alternative, rapid method consists of extracting phloem sap from bark samples via centrifugation (also illustrated in Figure 1) [46]. The centrifugation method is easy and allows one to collect a certain volume of undiluted phloem sap (thus solving issue (v); the unknown concentration and sap volume). However, the possibility of contamination by other cellular components cannot be overlooked, and this method requires high precision when separating tissues, in particular removing the xylem before centrifugation.

Phloem sap collection methods may impact the metabolic composition. The two recognized potential artifacts relate to sugars and amino acids. In particular, sucrose can be cleaved by enzymes to fructose and glucose during sampling, e.g., during exudation (discussed further below). Using the EDTA method, a higher glutamate concentration has been found compared to aphid stylectomy in *Solanum lycopersicum* [47]. This phenomenon may be due to the action of glutaminase (which cleaves glutamine into glutamate + NH_3_) during exudation. By contrast, no difference in amino acid composition has been found between EDTA and centrifugation methods in *Citrus sinensis* [45].

## 3. Phloem Sap Metabolites

Metabolic analyses carried out in the past decades with different techniques (summarized in Table 1) have shown that phloem sap is not just a concentrated sucrose/amino acid solution. In fact, many compounds of different metabolite families have been found, suggesting that phloem sap has metabolic functions beyond C, N and S redistribution from source leaves to sink organs. Nevertheless, it must be recognized that metabolites found in phloem sap depend on the analytical technique (typically, HPLC, GC-MS or LC-MS), and thus our current knowledge might not be strictly representative. Methods coupled to mass spectrometry allow analyses of many more metabolites than just HPLC, and thus GC-MS and LC-MS usually give access to a more representative picture of the metabolome. That said, GC-MS is usually dedicated to primary metabolites while LC-MS is more adapted to metabolites of higher molecular mass (such as secondary metabolites). In terms of concentration, current metabolomics techniques (such as GC-MS and LC-MS) are semi-quantitative and adapted to comparing samples rather than providing absolute concentrations (in mM). Unfortunately, the amount of sap that can be collected with current techniques is generally too small (a few µL) to allow quantitative analysis by NMR. This issue can be problematic since absolute concentrations are required to compute phloem transport rates (in mol metabolite s^−1^). In addition, some metabolites can interact with inorganic ions (e.g., some organic acids can coordinate, or precipitate with, divalent cations), and thus the knowledge of absolute concentrations can be useful. Interestingly, sap samples can also be used for protein and ribonucleoprotein complex analysis (polyacrylamide gel electrophoresis and mass spectrometry), via sap exudation after inflorescence puncture, as shown in *Brassica napus* [48].

In general, sugars are the major component of phloem sap, representing more than 70% of phloem sap metabolites. In herbaceous plants, sucrose concentrations range from 400 to 1400 mM [49], with some variation (maize (*Zea mays*) 900 mM, [50]; 844 mM, [51]; rice (*Oryza sativa*) 574 mM, [52]; barley (*Hordeum vulgare*) 1030 mM, [53]; wheat (*Triticum aestivum)* 251 mM, [54]; and castor bean (*Ricinus communis*) 270 mM, [55]). Likewise, sucrose concentration varies considerably between tree species from 65 mM to 1 M (for instance, oak (*Quercus robur*) ~1 M, [56]; beech (*Fagus sylvatica*) 790 mM, [57]; magnolia (*Magnolia kobus*) 850 mM, [57]; Eucalyptus (*Eucalyptus globulus*) 220 mM, [58]; and lemon tree (*Citrus limon*) 65 mM, [59]). It is believed that other soluble sugars such as fructose and glucose are also present in phloem sap, and sometimes at relatively high concentrations. Evidence for the occurrence of hexoses has been provided with reliable phloem sap sampling techniques, including stylectomy [53,56,60,61], the EDTA method [45,61,62,63,64,65], the incision method [66,67] and centrifugation [68]. Fructose and glucose content depends on species (e.g., found in lemon tree, wheat and maize [45,62] but absent in castor bean [21,55]) and developmental stage; for instance, it depends on grain filling stage, N fertilization and CO_2_ concentration in wheat [65,69,70]. When hexose concentration is high, it likely reflects the action of sucrose-cleaving enzymes during sampling (invertase, sucrose synthase) and/or the contribution of hexose from petiole tissues to the extract obtained by exudation [17,71]. Sugar alcohols (polyols) are also part of phloem sap composition, mannitol, sorbitol, inositol and xylitol being the three most common phloem sugar alcohols [34,62,64,67,68]. Mannitol concentration varies considerably between species, from 0.75 to 145 mM or even 400 mM in lemon trees, *Fraxinus excelsior* and celery (*Apium graveolens*), respectively [54,59,72]. In trees of the Rosaceae family, polyols are of importance: for example, sorbitol is more abundant than sucrose in phloem sap from *Prunus* sp. [68,72]. Inositol is also present in the phloem sap of many *Citrus* species, along with xylitol [45].

**Table 1:** Literature survey of papers where phloem sap metabolic analyses have been carried out (classified by sap sampling technique).

Sap Sampling Method	Analytical Technique	Species	Observed Metabolites	References
Bark sampling	GC-MS	*Picea abies*	Carbohydrates	[73]
Bark sampling	GC-MS	*Quercus cerris*	Carbohydrates, fatty acids and organic acids	[74]
Bark sampling and EDTA	GC-MS	*Vitis* sp.	Amino acids, carbohydrates, organic acids and sugar alcohol	[64]
Centrifugation	GC-MS	*Morus multicaulis*	Amino acids, carbohydrates, organic acids, phytohormones and sugar alcohol	[75]
Centrifugation	GC-MS	*Prunus* sp.	Amino acids, carbohydrates, organic acids and sugar alcohol	[68]
Centrifugation	Raman spectroscopy	*Quercus rubra*	Carbohydrates	[76]
Centrifugation	GC-MS	*Citrus* sp.	Amino acids, fatty acids, organic acids and sugar alcohol	[77]
Centrifugation	GC-MS	*Citrus* sp.	Amino acids, carbohydrates, fatty acids, organic acids, phytohormones, sugar acids and sugar alcohol	[59]
Centrifugation	GC-MS	*Citrus* sp.	Fatty acids	[78]
EDTA	GC-MS	*Arabidopsis thaliana*	Amino acids, carbohydrates, organic acids and sugar alcohol	[65]
EDTA	HPLC	*Arabidopsis thaliana* and *Sinapis alba*	Amino acids	[79]
EDTA	LC-MS	*Arabidopsis thaliana*	Amino acids, carbohydrates, fatty acids and organic acids	[80]
EDTA	HPLC	*Solanum tuberosum*	Amino acids and carbohydrates	[60]
EDTA	Chromatography	*Chenopodium rubrum*, *Perilla crispa* and *Pharbitis nil*	Carbohydrates and sugar alcohols	[44]
EDTA	Radio-labelling	*Anemone sylvestris*, *Centranthus ruber*, *Digitalis purpurea* and *Pulsatilla vulgaris*	Carbohydrates	[71]
EDTA	Automated amino acid analyser	*Picea abies*, *Fagus sylvatica*	Amino acids and carbohydrates	[81]
EDTA	GC-MS and UHPLC-FLD	*Plantago major* and *Poa annua*	Amino acids, carbohydrates, organic acids and sugar alcohols	[63]
EDTA	HPLC	*Solanum lycopersicum*	Amino acids	[47]
EDTA	GC-MS	*Zea mays*	Amino acids, carbohydrates, organic acids and sugar alcohols	[62]
EDTA and centrifugation	GC-MS	*Citrus sinensis*	Amino acids, carbohydrates, fatty acids, organic acids and sugar alcohols	[45]
EDTA and isotope composition	HPLC and automated amino acid analyser	*Fagus sylvatica*	Amino acids and carbohydrates	[82]
EDTA-HEPES	Plate reader (enzymatic assay)	*Solanum lycopersicum*	Carbohydrates	[83]
Incision	GC-MS	*Cucurbita maxima*	Amino acids, carbohydrates and organic acids	[66]
Incision	Chromatography, FCR and amino acid analyser	*Ricinus communis*	Amino acids, carbohydrates and phytohormones	[55]
Incision	Refractometry and amino acid analyser	*Nicotina glauca*	Amino acids and carbohydrates	[84]
Incision	HPLC	*Eucalyptus globulus*	Amino acids, carbohydrates and organic acids	[58]
Incision	HPLC and refractometry	*Ricinus communis*	Amino acids, carbohydrates and organic acids	[21]
Incision	GC-MS	*Cucumis sativus*	Amino acids, carbohydrates, organic acids and sugar alcohols	[67]
Incision	GC-MS	*Cucurbita maxima*	Fatty acids	[85]
Incision	Chromatography	*Robinia pseudoacacia*, *Quercus borealis*, *Quercus robur* and *Fraxinus americana*	Amino acids and carbohydrates	[86]
Stylectomy	Automated amino acid analyser	*Medicago sativa*	Amino acids	[87]
Stylectomy	HPLC	*Triticum aestivum*	Amino acids and carbohydrates	[54]
Stylectomy	HPLC and fluospectrometry	*Oryza sativa*	Amino acids, carbohydrates and nucleotides	[52]
Stylectomy	CE-LIF	*Arabidopsis thaliana*	Amino acids	[88]
Stylectomy	HPLC	*Zea mays*	Amino acids	[89]
Stylectomy	HPLC	*Brassica napus*	Amino acids and carbohydrates	[51]
Stylectomy	HPLC	*Plantago major*, *Plantago maritima*, *Prunus persica* and *Apium graveolens*	Carbohydrates and sugar alcohols	[72]
Stylectomy	HPLC	*Zea mays*	Amino acids, carbohydrates and nucleotides	[50]
Stylectomy	HPLC	*Quercus robur* and *Fraxinus excelsior*	Carbohydrates and sugar alcohols	[56]
Stylectomy	GC-MS	*Triticum aestivum*	Amino acids, carbohydrates and organic acids	[69]
Stylectomy	HPLC	*Trifolium pratense*, *Medicago sativa*, *Vicia faba* and *Pisum satiuum*	Amino acids	[90]
Stylectomy	HPLC	*Brassica napus*	Amino acids and carbohydrates	[91]
Stylectomy	Non-aqueous fractionation	*Hordeum vulgare*	Amino acids and carbohydrates	[53]
Stylectomy	Enzymatic assay and LC	*Beta vulgaris*	Amino acids and carbohydrates	[61]
Stylectomy	HPLC	*Oryza sativa*	Phytohormones	[92]
Stylectomy and ^11^C radiotracing	HPLC	*Zea mays*	Amino acids and carbohydrates	[93]
Stylectomy and EDTA	HPLC	*Fagus sylvatica*, *Magnolia kobus* and *Gnetum gnemon*	Carbohydrates and sugar alcohols	[57]
Stylectomy and EDTA	Chromatography, amino acid analyser and SDS-PAGE	*Lactuca sativa*	Amino acids and carbohydrates	[94]

Amino acids are the second most abundant metabolites in phloem sap, representing a small percentage of the total sap concentration with substantial variations between species, from about 5% to 15% [50,65,80], up to 360 mM in maize [62]. The prevalent amino acids are glutamate (Glu), glutamine (Gln), aspartate (Asp) and asparagine (Asn) [47,52,55,58,66,69,81,84,89,90]. However, in other species, other amino acids have been found to be more abundant: proline (Pro), alanine (Ala) or glycine (Gly) in *Arabidopsis*, *Citrus* species or maize [45,50,59,62,80]. Interestingly, amino acid phloem sap composition changes with the photoperiod. Under long days, Glu, Asp, and Ser decrease while Gln and Asn increase compared to short days, in *Arabidopsis* [79]. A similar change has been found in spruce (*Picea abies*) [81].

Organic acids are also present in phloem sap, at rather small concentrations, depending on the species. In *Arabidopsis*, total organic acid concentration is less than 0.5 mM [65], i.e., about 5% of the total metabolites. The same amount (5%) has been found in maize [62] and *Eucalyptus* (5 mM, [58]) but higher content has been found in others, e.g., castor bean (30 to 47 mM [55]) and lemon tree (44 to 232 mM, [59]). Most represented organic acids are from the tricarboxylic acid pathway: malate, citrate and aconitate [21,58,62,66]. Quinate has also been found in *Prunus* [68] and *Citrus* [59] species. In squash (*Cucurbita maxima*), lactate is as concentrated as malate [66], and propanoate and maleate are as concentrated as malate in *Arabidopsis* [80]. Organic acids are functionally important, not only for metabolism (oxaloacetate and fumarate metabolism, see below) but also for electroneutrality, carboxylate groups balancing positive charges carried by K^+^ (which is present at very high concentration in phloem sap). In other words, organic acid concentration should be related to ion species and are of paramount importance when anions availability (e.g., chloride, phosphate) varies. For instance, phosphorus availability impacts malate, succinate and citrate [67], while malate and fumarate are impacted by N supply [65]. These relationships are reminiscent of the malate/nitrate shuttle hypothesis, whereby nitrate assimilation is coupled to the biosynthesis of malate in leaves *(*i*)* to ensure electroneutrality, and *(*ii*)* to be sent via the phloem to roots, where it is decarboxylated, generating bicarbonate [95,96]. Additionally, in *Arabidopsis*, phloem sap malate and fumarate are positively correlated with raffinose, suggesting a link with galactose and inositol metabolism [65].

Free fatty acids are often overlooked in phloem sap composition. However, they may be present at a higher content than organic acids in most species tested so far. For example, fatty acids and their derivatives reach 13% of total metabolites in *Arabidopsis* [80]. Their concentration has been found to be up to 5 mM in *Citrus* species [45,59]. Palmitate and oleate appear to be prevalent fatty acids, along with stearate (*Citrus*, *Arabidopsis*) [45,78,80] and specific oxylipins, and phospholipids were detected only when the EDTA method was used (in *Arabidopsis*) [97]. It has been suggested that sap collection methods can change fatty acid composition or perhaps explain their presence in sap samples. Indeed, the centrifugation method can lead to fatty acid contamination from cell membranes. Therefore, the presence of fatty acids in phloem sap samples is not considered to be a definitive piece of evidence for fatty acid transport in SE [17].

Other compounds have been observed in phloem sap including hormones such as abscisic acid, auxin, gibberellins and cytokinins [55,75,92]. Polyamines are also commonly found in phloem sap, along with sulfur-containing compounds (S-adenosyl methionine (SAM), S-methyl methionine (SMM) and glutathione) and secondary metabolites including phenylpropanoids. S-containing compounds are important for (*i*) plant S nutrition and redistribution in species where sulphate reduction takes place in leaves and (*ii*) methylation (C_1_ metabolism) typically via the utilization of SAM and SMM.

## 4. Metabolic Pathways Reflected by Phloem Sap Metabolome

It is worth noting that the diversity of metabolites found in phloem sap (and summarized in Section 3 above) reflects different metabolic pathways such as sugar metabolism, nitrogen and sulfur assimilation and amino acid synthesis, the tricarboxylic acid pathway, arginine metabolism and polyamine synthesis. There is presently some uncertainty as to whether some of these pathways take place in phloem cells themselves (CC and SE) or only reflect source cell metabolism. It is well-accepted that major amino acids and sugars come from source cell N assimilation and photosynthesis, respectively. Specific amino acid synthesis may occur in phloem cells themselves such as asparagine since a phloem isoform of asparagine synthetase has been found in *Arabidopsis* [98,99]. Pioneering studies have shown that several enzymatic activities are absent from phloem sap in particular those involved in sugar cleavage and utilization [100]. This is consistent with the fact that phloem sugar transport should not compete with sugar catabolism. Accordingly, it is generally believed that SE mitochondria are small and not numerous probably reflecting limited catabolic activity [101].

Recently, proteomic analyses of phloem sap and phloem cells have also shown that many enzymes of primary C and N metabolism can be found, suggesting that many metabolites probably originate from phloem cell metabolism itself [15,102]. Surprisingly, this includes enzymes involved in sugar cleavage and utilization, as well as respiration. It strongly suggests that primary C metabolism in phloem cells is regulated post-translationally so as to avoid excessive sucrose degradation operating concurrently with sucrose transport. Interestingly, several enzymes of N metabolism are also found in phloem sap, indicating some potential for SE to modify amino acid composition or synthesize amino acid derivatives such as polyamines.

However, it is worth noting that, until now, nitrate reductase and APS (adenosine phospho-sulfate) reductase have not been found in phloem sap proteome, suggesting that SE (and perhaps, CC) are incapable of N and S reduction [102]. This is consistent with the fact that phloem sap movement can play a role in nitrate redistribution from source and senescing leaves to developing organs and roots, with nitrate molecules playing the role of both nutrient and signal (for a review, see [103]).

**Figure 2:**
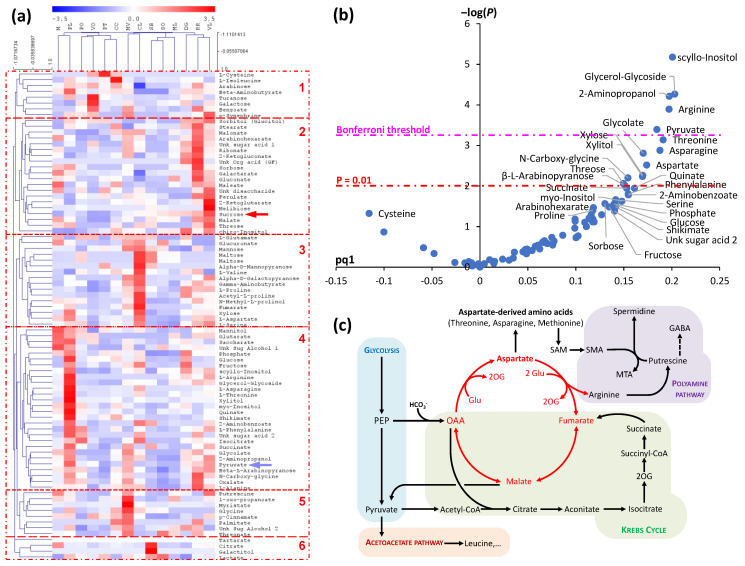
Metabolic analysis of phloem sap in *Citrus*. (**a**) Hierarchical clustering analysis showing covariation groups of metabolites when sap composition changes between cultivars. Sucrose and pyruvate are shown by red and blue arrows, respectively. (**b**) Volcano plot combining univariate (–log *p*-values) and multivariate (orthogonal partial least square (OPLS) loadings, pq1) analyses to identify best drivers of total phloem concentration (which increases from left to right). *p*-value thresholds (0.01 and Bonferroni) are shown with dash-dotted lines. (**c**) Simplified aspartate cycle (red) and its connections to other pathways. Abbreviations: 2-OG, 2-oxoglutarate; GABA, 4-aminobutyrate; Glu, glutamate; MTA, S-methyl thioadenosine; OAA, oxaloacetate; PEP, phosphoenolpyruvate; SAM, S-adenosyl methionine; SMA, S-adenosyl methioninamine. This figure has been generated by re-analysis of the original dataset in [59], using MeV (ANOVA) and Simca^®^ (OPLS).

GC-MS-based metabolomics analysis of phloem sap in *Citrus* cultivars has shown that many metabolite contents change along with the total sap osmolarity [59]. This indicates that the increase in total sap concentration is not simply associated with a general increase in loading capability of source leaves but is also associated with modifications in metabolic pathways. This is readily visible via combined univariate-multivariate statistics, with total sap concentration as a response variable (Figure 2). As phloem sap concentration changes, sucrose covaries with organic acids including malate (cluster 2, Figure 2a, red arrow). Even so, the amino acids hexoses and inositol are more correlated to the total concentration than sucrose (Figure 2b). Amongst amino acids, arginine, aspartate and asparagine are good markers of a high phloem sap concentration while cysteine appears to be a marker of low phloem sap concentration. Metabolites that increase concurrently with total phloem sap concentration include pyruvate and glycolate (cluster 4, Figure 2a, blue arrow). Such changes are reminiscent of the aspartate ‘cycle’ (illustrated in Figure 2c, in red) which connects organic acid metabolism (malate) to aspartate via arginine biosynthesis. Interestingly, the enhancement of the aspartate cycle (and the increase in hexoses) has been found to be associated with a low potassium availability in leaves [104], suggesting that phloem sap metabolome relates to nutrients, in particular K^+^ concentration. Perhaps coincidentally, a balance between sucrose and K^+^ concentration has been found in maize phloem sap [93]. Additionally, K^+^ concentration is believed to be essential to potentialize sucrose recapture by SE [105].

## 5. Phloem Sap Metabolites and Plant Resistance to Environmental Cues

It is now recognized that several phloem sap metabolites play a role in plant defense or resistance against pathogens. Using different *Citrus* cultivars, it has been suggested that tolerance to *Candidatus liberibacter asiaticus* is positively correlated to mannitol, phenylalanine, tyrosine and tryptophan [59]. In the case of aromatic amino acids, this correlation probably reflects the availability of precursors of phenylpropanoid secondary metabolites that play a role in disease resistance. Additionally, polyols appear to be linked to host acceptance of psyllid species feeding on *Prunus* species, *Malus domestica* or *Pyrus communis* (Rosaceae) [68]. In grapes (*Vitis vinifera*), changes in sap inositol content via specific scion-rootstock associations correlate to pathogen resistance [64].

Amino acid composition in phloem sap appears to respond to environmental factors such as nutrient availability. When supplied with high levels of nitrogen (N), Glu content increase in *Arabidopsis* phloem sap and conversely, Pro, Gln and γ-aminobutyrate (4-aminobutyrate; GABA) increase under low N availability [65]. Total amino acid concentration also changes with N supply as has been shown in canola [91]. Other conditions such as low phosphorus (P) availability and water deficit also affect amino acid composition [65,87]. Polyol content in phloem sap (such as sorbitol and erythritol) have also been found to impact drought and cold tolerance [59,62]. Recently, metabolomics analyses of cucumber plants subjected to phosphorus deficiency have shown important changes in phloem sap metabolome, not only in carbohydrates (galactitol, fructose) but also in organic acids (e.g., oxalate, citrate, fatty acids) along with nitrogenous compounds (e.g., ethanolamine, 4-aminobutyrate and pyroglutamate) [67]. Metabolomics analyses of phloem sap exudates during root waterlogging have found a change in the sugar to organic acid ratio, suggesting *(*i*)* a crucial role of the balance between loading in shoots and unloading in roots for sap composition and *(*ii*)* potentially, a negative feedback of metabolite accumulation in phloem sap onto shoot metabolism, such as sugar interconversions and respiration [106].

## 6. Phloem Sap Metabolome: Unforeseen Whole-Plant Metabolic Cycles?

**Figure 3:**
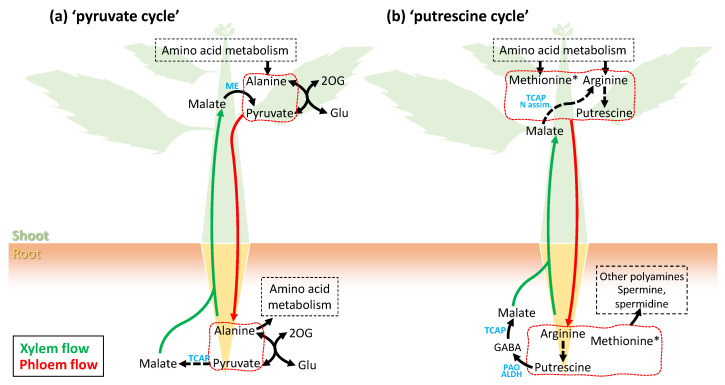
Two examples of possible metabolic cycles suggested by phloem sap metabolome. (**a**) Pyruvate cycle. Alanine and pyruvate are interconvertible via alanine transaminases with glutamate (Glu) and 2-oxoglutarate (2OG). They are present in phloem sap and could feed malate production in sink organs (roots) via the tricarboxylic acid pathway (TCAP), and malate can then be sent back to shoots via xylem sap. In shoots, malate can be cleaved by the malic enzyme (ME) to produce pyruvate. (**b**) Putrescine cycle. Arginine, methionine and putrescine synthesized in shoots can be used to feed higher degree polyamine synthesis (spermine, spermidine) in roots, and putrescine can be oxidized to 4-aminobutyrate (GABA) via polyamine oxidase (PAO) and aldehyde dehydrogenase (ALDH) and then to malate via the TCAP. As in (**a**), malate can be exported back to shoots to feed organic acid synthesis, and thus glutamate and aspartate production via N assimilation, sustaining arginine synthesis. The asterisk stands for the contribution of methionine derivatives: SMM and SAM.

The presence of many metabolites in phloem sap raises the question of their fate and role when they reach sink organs, including the roots. While the general principle of sugar and amino acid utilization is well-accepted (C and N redistribution from source leaves to sinks), uncertainty remains as to whether other metabolites are simply consumed or converted to derivatives that can be recycled back to shoots via xylem sap. It has been argued that a metabolic cycle between roots and shoots takes place with some amino acids exported by roots to shoots (including in non-legumes) [107,108]. It has also been shown that organic acids such as malate can be found in xylem sap at a significant concentration, partly explaining the CO_2_ release by shoots via the malic enzyme which produces pyruvate (in addition to CO_2_ liberation from dissolved CO_2_ and bicarbonate) [109,110]. Since many organic acids can be found in phloem sap, they could be converted to malate (via the Krebs cycle) in roots and then exported back to shoots via the xylem. It is also intriguing to find free pyruvate in phloem sap [59]. Its origin could be linked to either transamination activity (via alanine transaminase) or glycolytic activity in SE. Pyruvate could then be used by sink organs to resynthesize alanine or used by mitochondrial metabolism. Taken as a whole, a pyruvate cycle could take place at the whole plant scale, whereby *(i)* pyruvate reaching roots via the phloem sap could be recycled to organic acids such as malate; *(ii)* malate would then be exported back to shoots, where it could be partly cleaved to pyruvate and carbon dioxide (Figure 3a).

Other whole-plant metabolic cycles are plausible, for example involving polyamines and C_1_-metabolism. Methionine (and its derivatives SMM and SAM) could be used directly in sink organs as methyl and aminoethyl donors to synthesize secondary metabolites, such as bases (nucleotides) or polyamines, which can be sent back to shoots. Additionally, polyamines can be synthesized from arginine (in particular, putrescine can be synthesized directly from arginine decarboxylase). Aside its signaling roles [111], putrescine can also be converted to organic acids via polyamine oxidase, with evolved ammonium being reassimilated to glutamine and glutamate (Figure 3b). It is worth noting that in *Citrus* cultivars, cysteine appears to be negatively correlated to arginine (and other amino acids) (Figure 2b), suggesting that cysteine could be consumed by polyamine metabolism (in fact, cysteine is utilized by trans-sulphuration to yield methionine and thus is required to synthesize SAM, which is in turn required for polyamine metabolism).

## 7. Conclusions and Perspectives

Despite the crucial importance of phloem sap for plant metabolism and development, available data on phloem sap metabolome are rather limited. The hurdle in phloem metabolome exploration is sap collection since phloem sap samples are in very small amount (low volume) or might be adulterated by sampling methods themselves. New generation instruments associated with high sensitivity and high mass resolution (for example, GC coupled to high resolution mass spectrometry with the Orbitrap^®^ technology) will probably give access to more comprehensive sap metabolic profiling in the future. Additionally, alternative sampling strategies have to be found to enable pure, unadulterated phloem sap collection. Single-cell analysis, already used for proteomics, is very promising in that regard: in the case of phloem biology, a recent study has shown how to isolate sieve tubes from bulk phloem to carry out proteomics analyses [15]. In terms of physiology, metabolic cycles can be hypothesized (such as those suggested in Section 6 above), but it should be recognized that there is presently little evidence, such as labelling with stable isotopes (^13^C, ^15^N or ^34^S) followed by isotope-assisted metabolomics. Future studies are desirable to resolve phloem sap metabolome and thus to better understand how phloem sap metabolism participates in plant responses to environmental conditions. Additionally, phloem sap analysis (exudation method) in maize has shown some link between sap metabolome and yield [62]. In the future, it would be of great interest to assess whether phloem sap biomarkers of crop performance can be identified.

## Supplementary Materials

The following supporting information can be downloaded at: https://www.mdpi.com/article/10.3390/ijms24086917/s1.
