# Supplementary material for: Phloem Sap Composition: What Have We Learnt from Metabolomics?
Source: Int J Mol Sci. 2023 Apr 7;24(8):6917. doi: 10.3390/ijms24086917 (PMC10139104; doi:10.3390/ijms24086917)
Supplement: Supplementary file 1 [file ijms-24-06917-s001.zip › ijms-2220290-supplementary.pdf]

Supplementary material

Table S1. Terms associated with phloem sap.

Term	Strict definition	Common utilisation and remarks
Phloem sap	Refers to total cell sap contained by phloem tissue, including SE, CC and phloem parenchyma cells.	In common parlance, ‘phloem sap’ only refers to the fluid contained by SE and moving between organs. This meaning is used in this paper.
Collected phloem sap	Refers to the fluid collected from phloem tissues, regardless of whether it is via stylectomy, exudation or other techniques.	This fluid is generally assumed to be representative of the fluid circulating in SE, although it can be contamined by xylem sap, or reflect either circulating phloem fluid or phloem sap stricto sensu (total cell sap).
Circulating phloem fluid (‘true’ sap)	Refers to the fluid in movement within SE.	Often considered equivalent to “phloem sap”. A synonymous term would be “sieve tube sap”.
Phloem sap circulation	Referes to the process by which the phloem fluid moves in SE and redistributes metabolites and water. This is a mass transfer phenomenon, with a fluid velocity, usually measured in cm s^{-1}.	The term ‘circulation’ in its broad sense is also useful to suggest *(i)* the complicated nature of the movement of phloem fluid between source leaves, developing leaves, roots and other sink organs; *(ii)* the possible occurrence of metabolic cycles whereby both phloem and xylem allow (re)circulation (i.e., cycling) of metabolites.
